# Minimally invasive supratentorial neurosurgical approaches guided by Smartphone app and compass

**DOI:** 10.1038/s41598-021-85472-3

**Published:** 2021-03-24

**Authors:** Bruno Fernandes de Oliveira Santos, Daniel de Araujo Paz, Victor Miranda Fernandes, José Calasans dos Santos, Feres Eduardo Aparecido Chaddad-Neto, Antonio Carlos Sobral Sousa, Joselina Luzia Menezes Oliveira

**Affiliations:** 1 Health Sciences Graduate Program, Federal University of Sergipe, Aracaju, SE Brazil; 2 Department of Neurology and Neurosurgery, Universidade Federal de São Paulo, São Paulo, SP Brazil; 3 Federal University of Paraíba, João Pessoa, PB Brazil; 4 Unimed Sergipe Hospital, Aracaju, SE Brazil; 5 Department of Internal Medicine, Federal University of Sergipe, Aracaju, SE Brazil; 6 Division of Cardiology, University Hospital, Federal University of Sergipe, Aracaju, SE Brazil; 7 Clinic and Hospital São Lucas / Rede D`Or São Luiz, Aracaju, SE Brazil; 8 Department of Neurosurgery, Hospital de Cirurgia, Aracaju, SE Brazil

**Keywords:** Neurological disorders, Medical imaging

## Abstract

The precise location in the scalp of specifically planned points can help to achieve less invasive approaches. This study aims to develop a smartphone app, evaluate the precision and accuracy of the developed tool, and describe a series of cases using the referred technique. The application was developed with the React Native framework for Android and iOS. A phantom was printed based on the patient's CT scan, which was used for the calculation of accuracy and precision of the method. The points of interest were marked with an "x" on the patient's head, with the aid of the app and a compass attached to a skin marker pen. Then, two experienced neurosurgeons checked the plausibility of the demarcations based on the anatomical references. Both evaluators marked the frontal, temporal and parietal targets with a difference of less than 5 mm from the corresponding intended point, in all cases. The overall average accuracy observed was 1.6 ± 1.0 mm. The app was used in the surgical planning of trepanations for ventriculoperitoneal (VP) shunts and for drainage of abscesses, and in the definition of craniotomies for meningiomas, gliomas, brain metastases, intracranial hematomas, cavernomas, and arteriovenous malformation. The sample consisted of 88 volunteers who exhibited the following pathologies: 41 (46.6%) had brain tumors, 17 (19.3%) had traumatic brain injuries, 16 (18.2%) had spontaneous intracerebral hemorrhages, 2 (2.3%) had cavernomas, 1 (1.1%) had arteriovenous malformation (AVM), 4 (4.5%) had brain abscesses, and 7 (7.9%) had a VP shunt placement. In cases approached by craniotomy, with the exception of AVM, straight incisions and minicraniotomy were performed. Surgical planning with the aid of the NeuroKeypoint app is feasible and reliable. It has enabled neurological surgeries by craniotomy and trepanation in an accurate, precise, and less invasive manner.

## Introduction

Less invasive and less morbid approaches require precise methods of locating points on the scalp surface based on medical images. Classically, craniotomy planning is based on the knowledge of craniometric landmarks and their assumed relationships with the underlying sulci and gyri, with a consequent inference of functionality.

It has long been attempted to locate points overlying intracranial lesions on the skull surface from axial computed tomography (CT) scans, either with the aid of multiple radiographs^1–3^, based on anatomical references^4^, or by using radiopaque markers^5,6^. Not infrequently, they are techniques of variable precision that require specific protocols and/or demand time beyond what is desired in daily practice.

Nowadays there are devices capable of demonstrating predetermined points on the cranial surface with millimetric accuracy; among the most used are the neuronavigation techniques^7^. However, such a technology has a relatively high cost and requires image acquisition in specific protocols. In some situations, the identification of a single point is sufficient for a satisfactory and less invasive approach. In countries with healthcare financing limitations, developing low-cost alternatives is crucial.

Some researches^8–15^ have already described methods to use open-source software in order to perform image-guided surgery without using the standard neuronavigation systems. The technique we intend to develop and evaluate in this work does not require expensive equipment and can be performed by the surgeon himself using his smartphone. Therefore, this study aims to develop a smartphone app in order to improve neurosurgical approaches, evaluate the precision and accuracy of the developed tool, and describe a series of cases using the referred technique.

## Materials and methods

### Image exam

The magnetic resonance (MR) images of the skull were obtained in a MAGNETON Sonata 1.5 T device (Siemens Healthcare, Erlangen, Germany), with an eight-channel head coil and having the following technical specifications: gradient of 40 mT/m, matrix of 256 × 256 pixels, field of view (FOV) of 256 × 256 mm and slice thickness of 1 mm. The T1 sequence with gadolinium was acquired in the sagittal plane with TR of 2000 ms and TE of 3.42 ms. The skull CT images were obtained with the Brilliance CT 64 System (Philips, 2004) with collimation of 20 × 0.625, 0.348 pitch, 512 × 512 matrix, 200 mm field of view, 140 kpV, 278 to 600 mA and 1 mm thick. The image files in the DICOM format were imported into the Horos 1.1.7 software (GNU General Public *License, version 3*). The “point tool” provides the three-dimensional coordinates (x, y, z) of each of the marked points.

Point A: Point of interest marked in the software.

Points R1 and R2: These points must correspond to two surface anatomical references easily identified in the patient (Example: *nasion* and external acoustic pore).

### NeuroKeypoint app

The *NeuroKeypoint* app was developed with the framework React Native for Android and iOS platforms. We used the standard Euclidean geometry formula of distance between two points in a three-dimensional space^16^ D^2^ = (x_1_ − x_2_)^2^ + (y_1_ − y_2_)^2^ + (z_1_ − z_2_)^2^, where D is the distance between two points, and (x_1_, y_1_, z_1_) and (x_2_, y_2_, z_2_) are their coordinates. With these data, the app calculates the distance between points A and R1 (R), and between A and R2 (r).

### Marking the point of interest in the scalp

**Figure 1:**
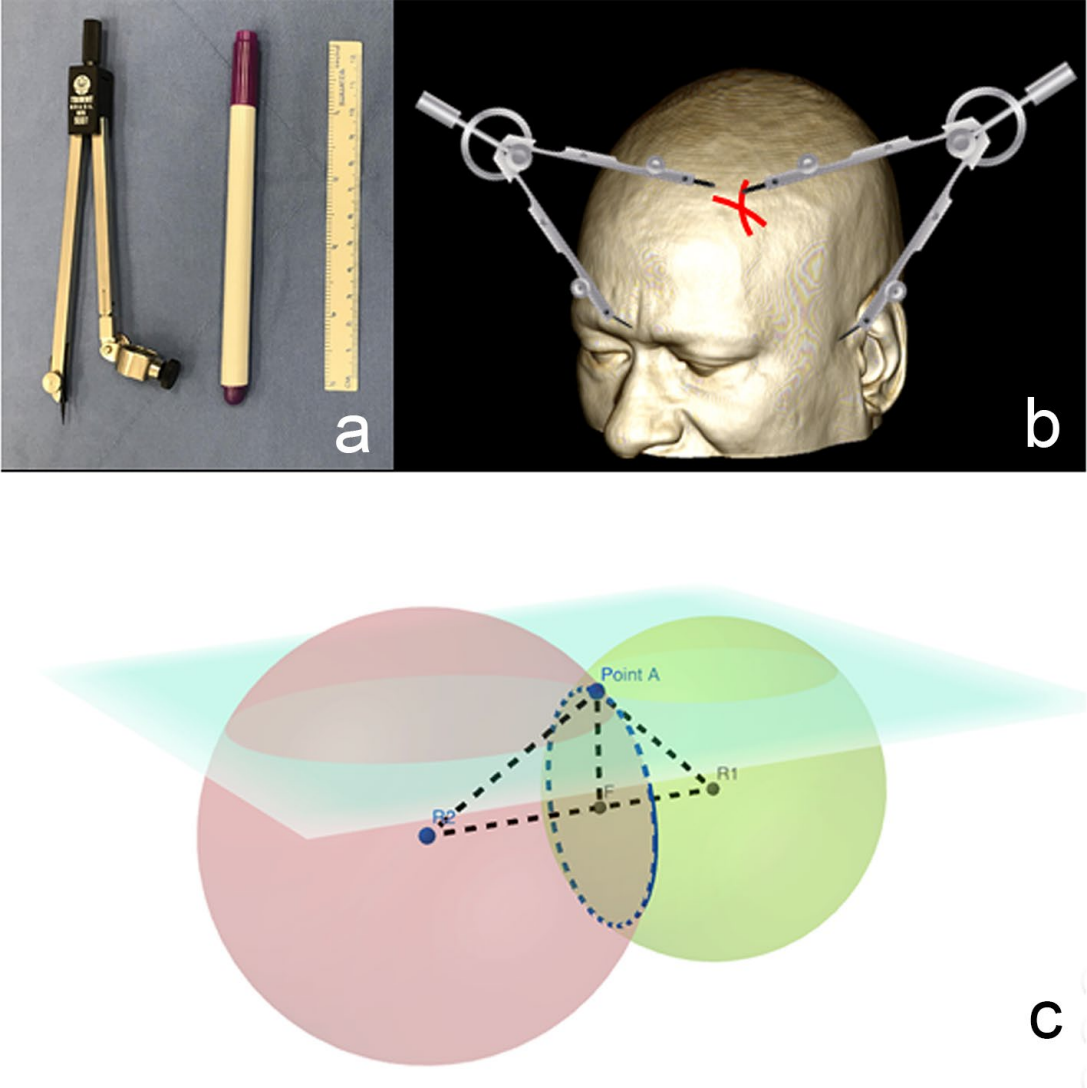
(**a**) Trident compass model 9001, surgical skin marker pen and ruler; (**b**) Demonstration of the marking of point A; (**c**) Geometric fundamentals.

The point of interest is marked based on the reference points that are easily identifiable both in the patient and in the medical image, similar to the technique previously described ^17^. Three-dimensional coordinates (x, y and z) of each of the points marked (target and references) were taken from Horos software and they were typed in the application. The two distances provided by the app correspond, one at a time, to the opening of the legs of the Trident model 9001 compass (Fig. 1a). A surgical skin marker pen is mounted on the compass. The steady compass leg is fixed at the corresponding reference point and an arc is drawn. After tracing the two arcs, the point of intersection between them will correspond to point A (Fig. 1b). Regarding geometric fundamentals, considering each of the two calculated distances (R and r) as the radius of two distinct spheres, we will have two spheres’ surfaces with an intersecting circle. The intersection between this circle and the scalp surface corresponds to only one point (point A), which is the point of interest (Fig. 1c).

### Precision and accuracy assessment

**Figure 2:**
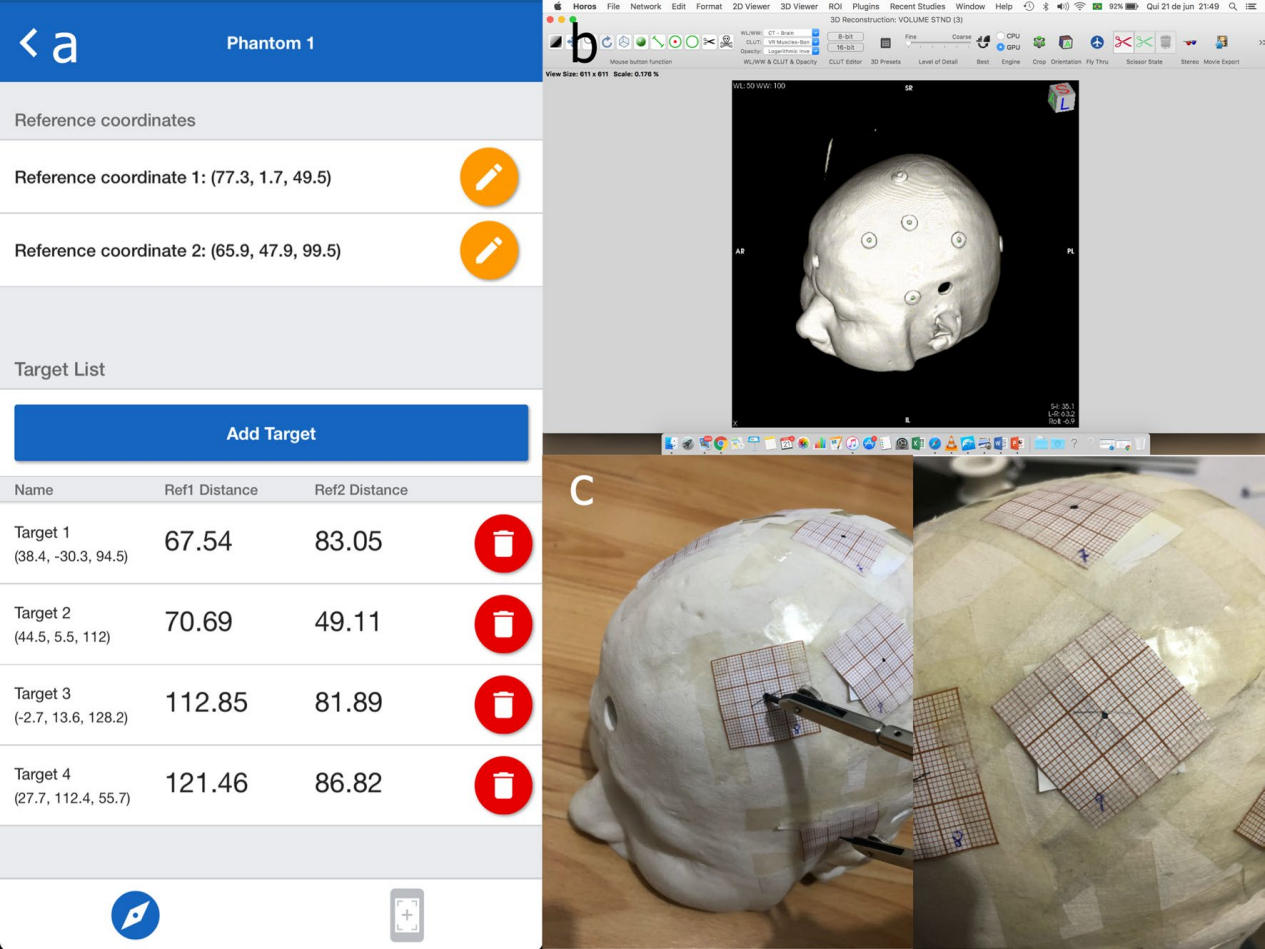
(**a**) Neurokeypoint app screenshot; (**b**) Horos 1.1.7 software screenshot showing the 3D reconstruction based on phantom CT scan with self-adhesive fiducials; (**c**) the app returned the measurement of the opening of the compass in order to mark an "x" at the point of interest over phantom surface.

A normal skull CT was randomly selected. A head phantom was printed from this CT image on a Z-Corp Model 310 Rapid Prototyping System. 12 self-adhesive fiducials were attached to the phantom’s surface and a phantom CT scan was acquired. Coordinates of the two reference points and of the 12 fiducials were then obtained. The app returned the measurement of the opening of the compass in order to mark an "x" at the point of interest (Fig. 2). Two independent neurosurgeons marked the 12 points each. The main error metric for neuronavigation systems is the target registration error (TRE), which, in this case, is the distance between the marked point and the intended target. We calculated the TRE using a millimeter paper, where the center corresponds to the target (fiducial center).

### Patients

This is a convenience sample, with prospective data collection. The patients who participated in the study were those who were submitted to neurosurgical treatment from September 2016 to June 2020 and who underwent adequate preoperative image examination for surgical planning. The exclusion factor was the unavailability of the DICOM file. The project was approved by the Ethics and Research Committee.

### Statistical methodology

We used mean TRE as the measurement of accuracy, as well as the dispersion measure for assessing precision. The systematic error assessment was calculated using a Bland Altman plot. Data analysis were performed using the Statistical Package for the Social Sciences (SPSS), version 20 (Chicago, IL, USA).

### Ethics approval

All procedures performed in studies involving human participants were in accordance with the ethical standards of the institutional and/or national research committee and with the 1964 Helsinki declaration and its later amendments or comparable ethical standards. The study was approved by the Intitutional Review Board of Universidade Federal de São Paulo (CAAE 57546416.6.0000.5505).

### Informed consent

Informed consent was obtained from almost all individual participants included in the study. Those not capable of giving their own consent due to decreased level of consciousness were also included in the study if written informed consent was obtained from the respective legal guardians.

### Consent for publication

The authors affirm that human research participants provided informed consent for publication.

## Results

**Figure 3:**
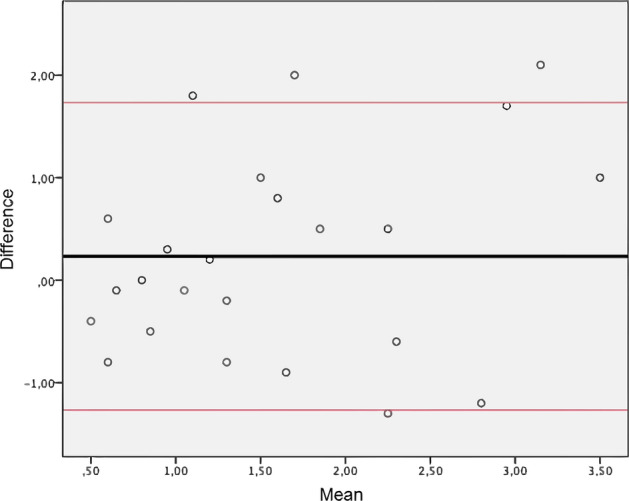
Bland Altman plot (inter-evaluators).

The app was successfully developed for both Android and iOS, and it maintained a stable operation on both mobile platforms. Both evaluators marked the frontal, temporal, and parietal targets with a difference of less than 5 mm from the corresponding intended point, in all cases, without statistically significant difference between locations. The overall mean TRE was 1.6 ± 1.0 mm. The mean accuracy was 1.5 ± 0.8 for neurosurgeon 1 and 1.7 ± 1.1 mm for neurosurgeon 2, with no statistically significant difference between them (*p* = 0.458). There was also no relevant difference between the test and the re-test (1.7 ± 1.1 vs 1.4 ± 0.9, *p* = 0.340). The use of fiducials in clinical cases can improve accuracy since it minimizes an important source of variability, which is the identification of reference points. The Bland Altman graph allows assessing the absence of systematic error among the evaluators, as well as the acceptable limit of the agreement (Fig. 3).

**Figure 4:**
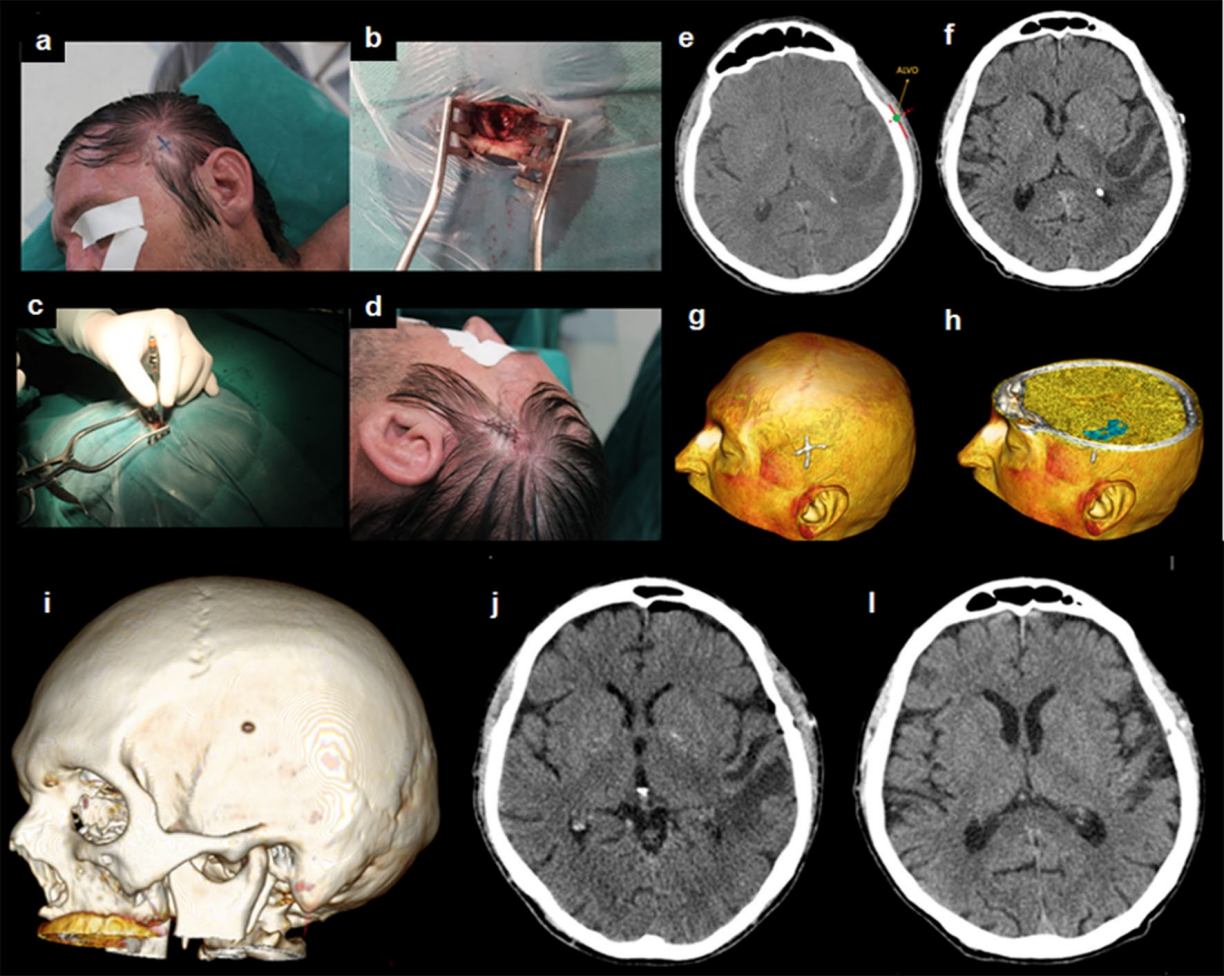
A 58-year-old man with a diagnostic hypothesis of left temporal brain abscess submitted to drainage through trepanation. (**a**), (**b**), (**c**), and (**d**) Intraoperative images showing the surgery step-by-step; (**e**) Surgical planning (**f**) Cranial CT scan after targeting; (**g**) and (**h**) pre-operative three-dimensional virtual reality reconstruction (3DVR) showing the target and its relationship with the lesion; (**i**) postoperative 3DVR with the trepanation; (**j**) Immediate postoperative cranial CT scan; (**l**) late radiological control, after 6 weeks, with complete resolution of the abscess.

**Figure 5:**
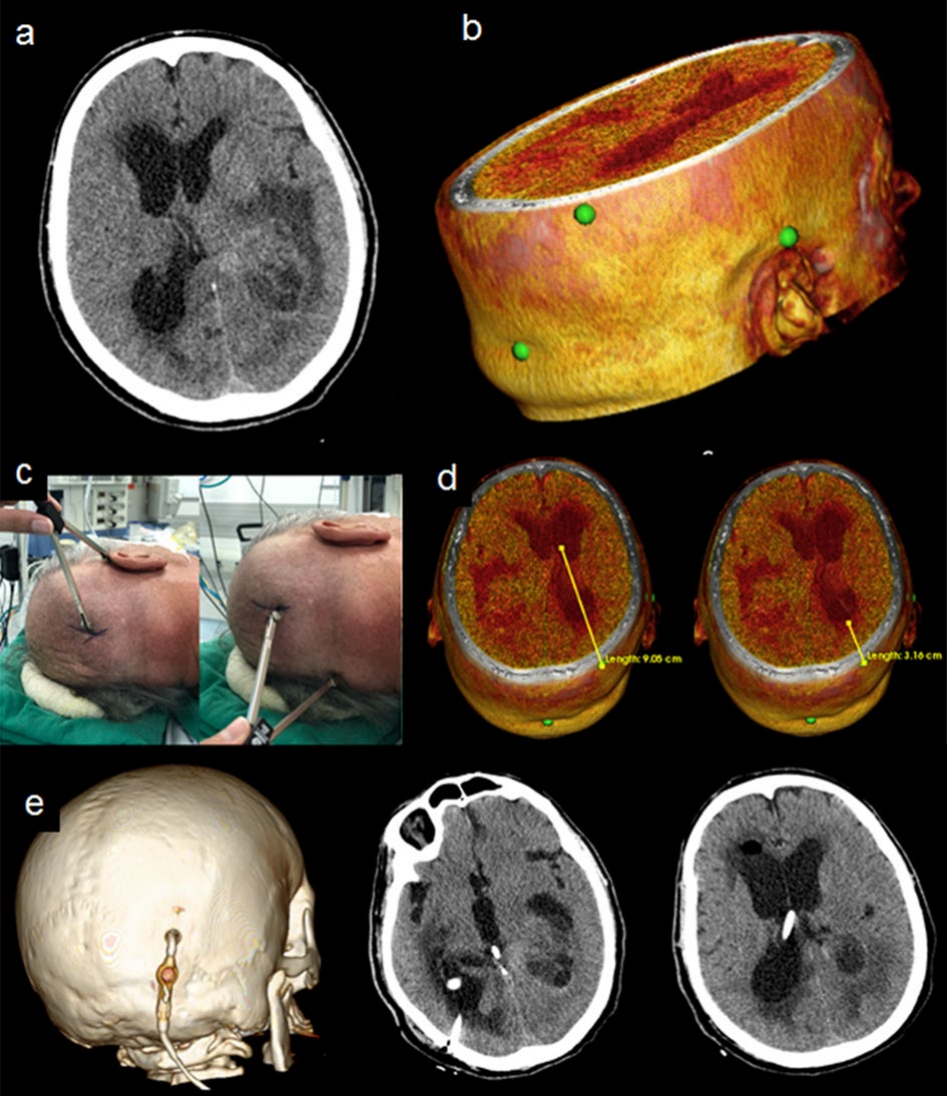
A 76-year-old patient diagnosed with an intraventricular tumor and hydrocephalus underwent VP shunt placement. (**a**) Cranial CT scan with a left intraventricular tumor and hydrocephalus; (**b**) 3DVR with the selected reference points and the target represented by green points; (**c**) image sequence showing head positioning and target identification using the compass (the target is represented by the intersection between the two lines drawn with the compass); (**d**) Surgical planning: distance between the target and the anterior horn of the right lateral ventricle and between the target and ventricular interior; (**e**) postoperative result.

A total of 88 patients were operated (Table 1) by authors (BFOS or DAP). First, the neurosurgeon (BFOS or DAP) marked the intended point using the app and the compass. Then they accepted or not the suggested location. In all cases, it was possible to locate the target in an anatomically plausible location. The neurosurgeons accepted and actually used the proposed location by the app in all cases. In ten cases, a neuronavigation system was available and it was used to check the target marked by the app. In all cases, neurogavigation system agreed with app target. Surgical planning of trepanations for VP shunts and drainage of abscesses was performed. In abscess cases, the target was selected based on the shortest path to the lesion through a small trepanation (Fig. 4). In cases of VP shunt placement, the point of interest on the scalp surface corresponded to the point having the shortest path to access the ventricle (Fig. 5). Once defined the entry point, catheter was placed using standard technique.

**Table 1:** Operated patients.

	n	Male	Mean age	Right side
Tumors	41	24 (58.5%)	50.0 ± 13.4	23 (56.1%)
Trauma	17	16 (94.1%)	50.2 ± 20.1	10 (58.8%)
ICH	16	7 (43.8%)	60.7 ± 10.1	8 (50.0%)
VP shunt	7	5 (71.4%)	49.8 ± 23.2	6 (85.7%)
Abscess	4	4 (100%)	54.2 ± 2.6	2 (50.0%)
Vascular	3	3 (66.7%)	44.7 ± 6.4	3 (66.7%)
Total	88	57 (64.8%)	52.0 ± 15.4	50 (56.8%)

**Figure 6:**
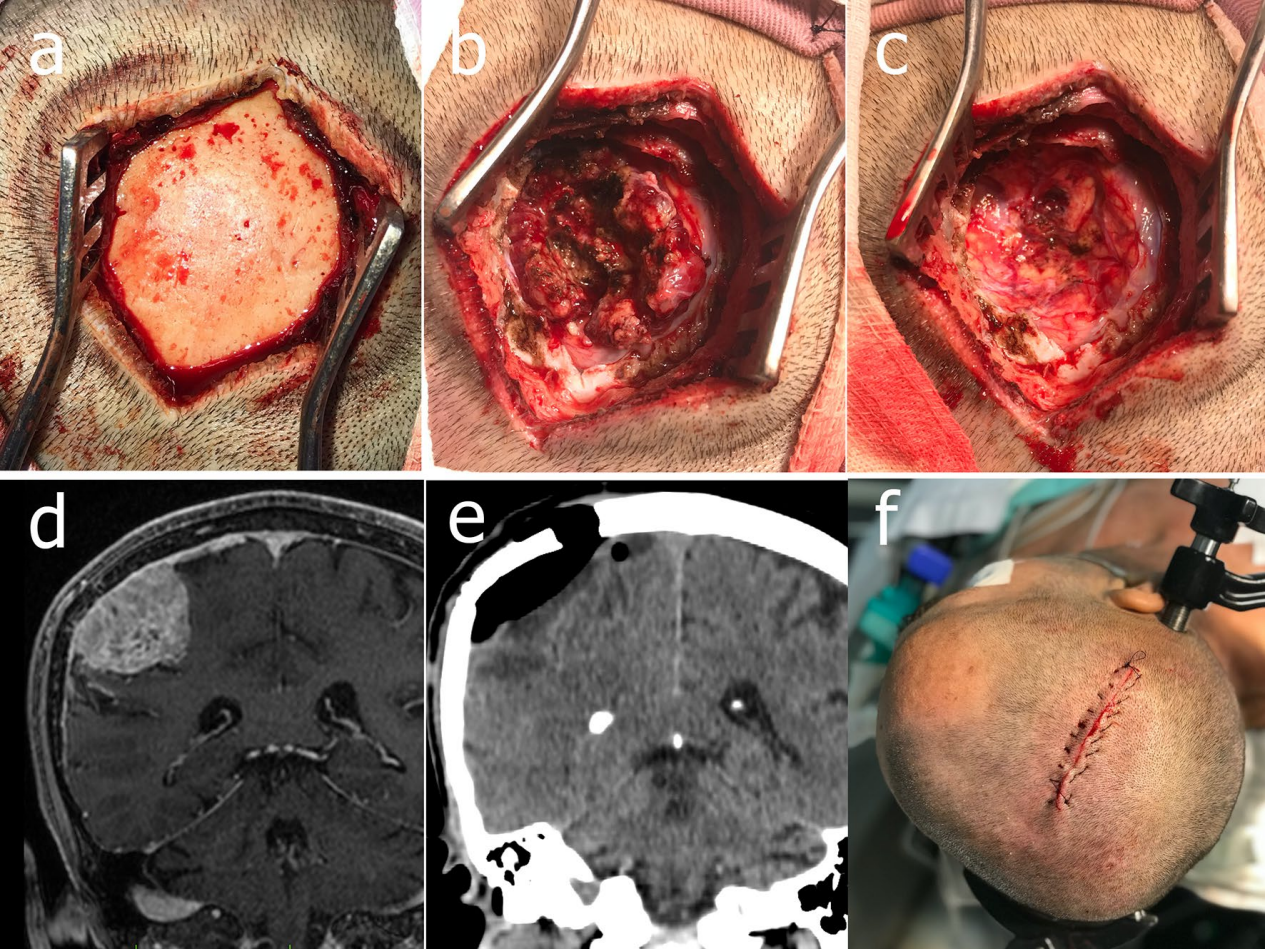
A 60-year-old man with left arm focal seizure diagnosed with a right parietal extra-axial mass lesion. (**a**) Straight incision initial exposition (**b**) circumferential tumor exposition after the removal of dura-mater; (**c**) post total resection appearance; (**d**) preoperative cranial MRI with the right parietal tumor; (**e**) postoperative cranial CT scan; (**f**) straight incision final appearance.

**Figure 7:**
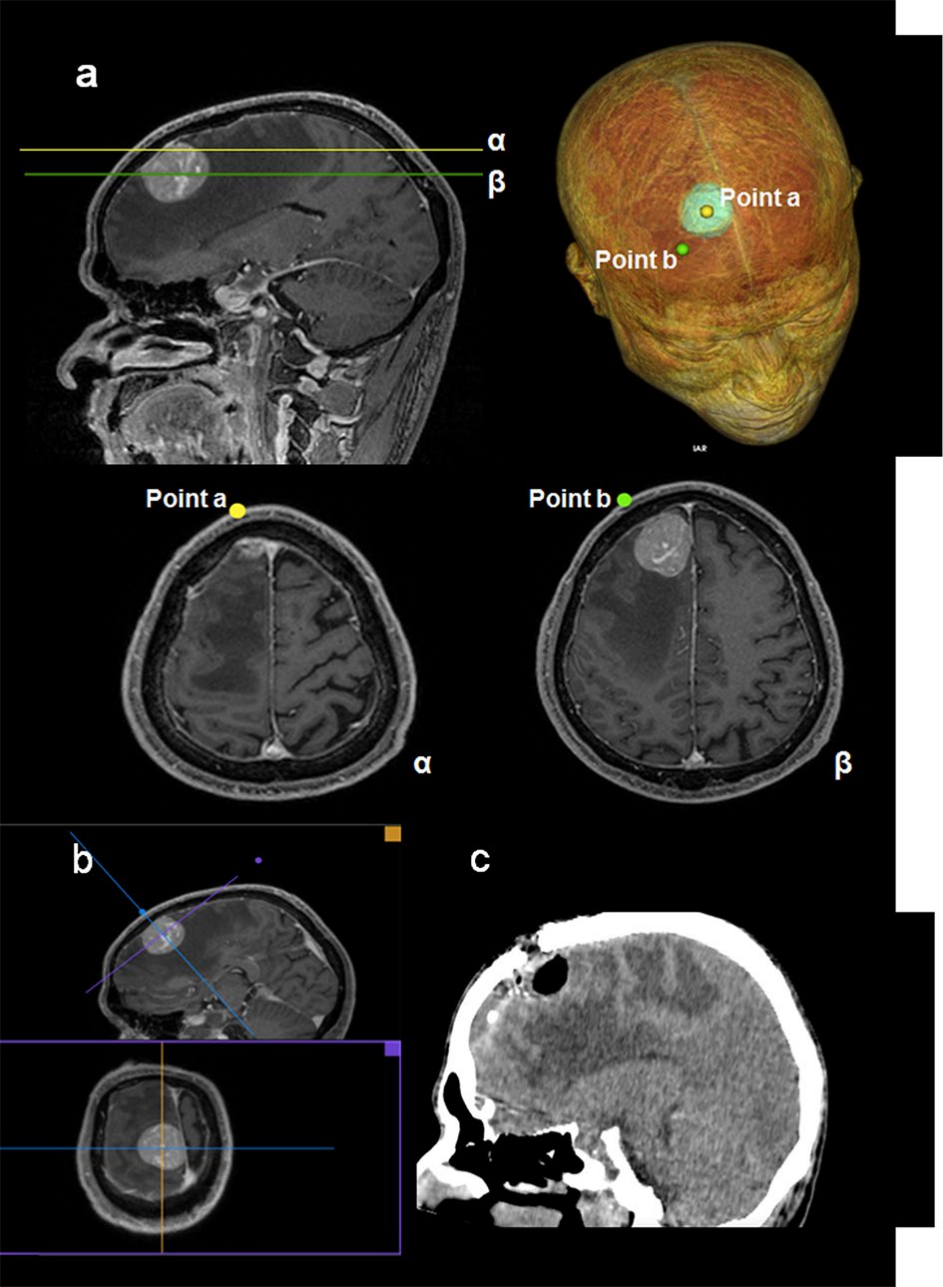
Demonstration of the importance of multiplanar reconstruction in a case of superior frontal gyrus tumor. A 58-year-old man presented to the hospital because of left hemiparesis. (**a**) Cranial MR image demonstrating two slices (α and β) with two options of target points (**a**, **b**), as well as 3DVR showing the points and the tumor; (**b**) multiplanar reconstruction perpendicular to the intended approach axis; (c) postoperative cranial CT scan demonstrating tumor gross total resection. A linear right frontal incision and a right frontal minicraniotomy were performed and the patient evolved with neurological improvement.

Craniotomies for meningiomas, gliomas, brain metastases, intracranial hematomas, cavernomas, and arteriovenous malformation were planned. The authors operated 41 tumor: 24 gliomas, 13 metastases, and 4 meningiomas. The target point was defined as the center of the intended craniotomy. With the exception of AVM, straight incisions and minicraniotomies were performed for all cases that were not approached by trepanation (Fig. 6). As a rule of thumb, craniotomies were designed in a circular shape centered on the target point in order to include the entire lesion. The incision was also centered on the target point and planned to be 1.5 times the diameter of the desired craniotomy. Mean craniotomy diameter was 6.4 ± 1.4 cm, ranging from 4.1 to 9.6 cm. Mean skin incision length was 9.7 ± 2.0 cm, ranging from 6.2 to 13.9 cm. Multiplanar reconstruction planning are recommended as axial slices are not reliable for defining the best craniotomy location and may lead to misplacing the target point (Fig. 7). The non-conventional planes demonstrated in Fig. 7b are not essential to point definition, but we advocate that they are important for an adequate surgical planning. Placing the target accurately in an inadequate point will result in an inadequate approach.

**Figure 8:**
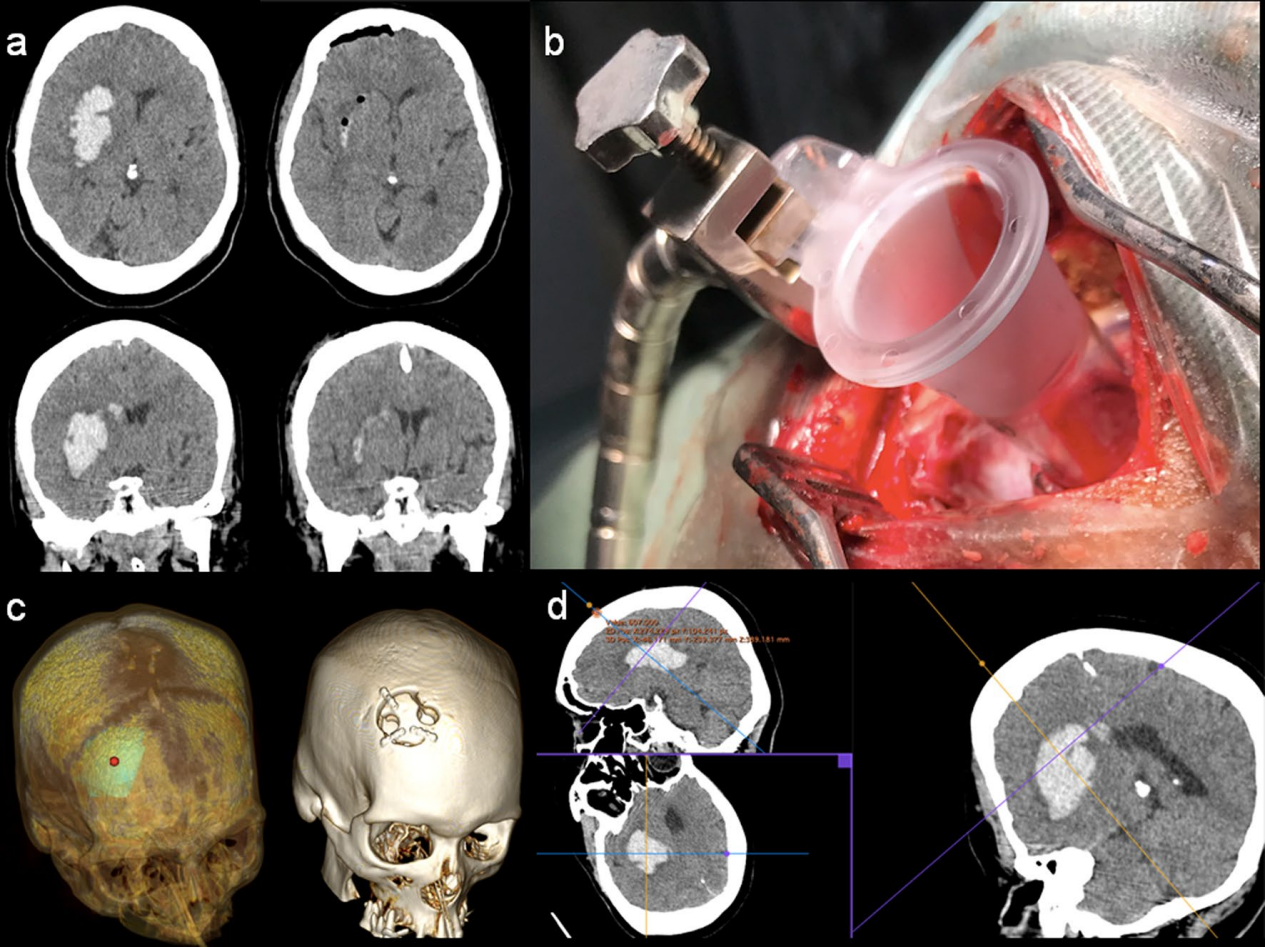
A 54-year-old woman with hypertension was admitted to the emergency room with a decreased level of consciousness and left hemiparesis. (**a**) Pre- and postoperative aspect of intracerebral hemorrhage in the right basal ganglia region (**b**) tubular approach through a small craniotomy preserving the brain tissue as much as possible; (**c**) 3DVR showing the surgical planning target point and craniotomy; (**d**) multiplanar reconstruction with “probe-eye view” in the purple square.

Deep lesions were planned to take into account a presumed non-functional route and an approach axis preferably perpendicular to the scalp surface. Tubular retractors were used in order to minimize surgical-related brain injury (Fig. 8).

## Discussion

The method proposed in this work was developed based on a problem of daily practice—the need for performing computer-assisted surgery, even with few resources. When it comes to low- and middle-income countries, in numerous cases, tools such as the neuronavigation systems are not available. An app that allows the precise demarcation of scalp targets at low cost was developed on Android and iOS platforms using both CT and MR imaging. The application was used in the surgical planning of trepanations for VP shunts and drainage of abscesses and also in the definition of craniotomies for meningiomas, gliomas, brain metastases, intracranial hematomas, cavernomas, and arteriovenous malformation.

Since the late nineteenth century, mechanical devices and mathematical concepts were merged in order to access a specific target in the brain ^18^. Earlier tools, in a pre-imaging epoch, used spherical coordinates such as Kocher’s craniometer ^19^ and Zernov’s encephalometer ^20^. The tricoordinate cartesian system represents an evolution and it is the foundation of stereotaxy ^21^. The method proposed in this paper combined these two concepts (spherical coordinates and cartesian space), the benefits of digital medical imaging, and the facilities of calculus in smartphones.

The accuracy of cranial access markings based exclusively on craniometric references is frequently criticized, and, in practice, limits the possibility of making less invasive approaches without the aid of tools such as neuronavigators. Previous work already pointed out a non-negligible margin of error that occurs when marking craniotomies without supplementary tools ^22^, especially if we consider planning for a minimally invasive approach.

Previous work ^23^ described a series of cases using a technique capable of assisting in the intraoperative localization of intra-axial lesions. In the same way, researchers ^24^ reported a vessel-to-vessel registration where the cortical veins were used as a surgical map. However, despite assisting microsurgery, these methods start from the point where the brain is already exposed, and they are not able to assist in the craniotomy planning. In this context, another paper ^17^ described a method using a compass and triangulation principles for locating points from a series of parallel two-dimensional images. Despite being easy to understand, the method requires calculations that may not be easy to perform at the bedside. In addition, the spacing between images in the printed film limits the precision of the technique.

Other researches already tried to develop easy to use solutions with smartphones, most of them with the aid of augmented reality ^25–29^. These methods involve manual adjustment of a projected virtual image, which can be difficult especially in a surgical position. More robust alternatives have already been described ^30–35^, and despite some of them being open-source, the implementation of these solutions is not a trivial task and demands specialized hardware. Despite the simplicity of the method presented in this work, the application allowed calculations to be performed quickly at the bedside. These calculations would require more time than that tolerated in the operating room environment. In addition, it proved to be ergonomically favorable, since it did not require any changes in the layout of the operating room.

The proposed technique had an overall accuracy of 1.6 ± 1.0 mm, which is acceptable. Previous study^36^ evaluated the accuracy of two neuronavigation systems using, for this, a phantom with a deformable surface that simulated the skin and obtained results of less than 1.5 mm for both systems. Other researchers ^37^ studied the influence of the registration technique on accuracy. They found values of 3.49 mm for ones that used adhesive fiducials, 3.96 mm for those based on anatomical references, and 3.33 mm for ones based on surface calibration. It must be considered that the studies have methodological nuances that could raise questions about comparisons between casuistry. However, it is intuitive to think that the method of this work is accurate and precise enough to justify its use alone or in association with other technologies. Besides, the performance of less invasive craniotomies with straight incisions was possible, which can be useful even in cases using the keyhole concept ^38^.

Planning surgical interventions is a demanding task. The surgeons rely on their sensorimotor, cognitive, and spatial abilities to perform mental transformation and to visualize medical images onto the patients’ surface. Concerning lesion location, when it comes to superficial ones, surgical planning is straightforward; however, deep lesions are challenging. Usually, we opt for targets in the scalp that provide perpendicular routes until the lesion. Approaches perpendicular to a plane tangent to the skull surface are more easily performed in a freehand manner than other angles. Tubular retractors minimize surgical-related brain injury. One of the technical pearls of utmost importance is the use of a multiplanar reconstruction feature. All the cases described in this work take advantage of this resource. It allows users to generate images from additional perspectives. One of the most important reconstructions is the one perpendicular to the intended approach. Such a set of images include the “Probe’s eye” view, which makes an orientation similar to that of the procedure possible. Taking multiplanar reconstruction into consideration could help avoid mistakes in surgical planning, as is demonstrated in Fig. 7.

In low- middle-income countries, especially where there is limited availability of technological resources for image-guided surgeries, implementation of this method should be considered. However, the application of this method in daily practice is highly dependent on the local reality peculiarities. Regarding the DICOM viewer software, Horos is not the only option. Any computer with a three-dimensional coordinate determination tool can be used, including the workstation that acquires the images. Nevertheless, using software with the option of multiplanar and oblique reconstructions is mandatory, as already mentioned.

If we consider that in 40% of the cases neuronavigation is used only for the demarcation of the craniotomy^39^, we will conclude that a good portion of the cases may benefit from this method. In this casuistry, the target point was defined as the center of the intended craniotomy. In other words, the definition of only one point was enough. In addition, the technique was ergonomic, since it was not necessary to change the regular operating room layout nor the habitual surgical workflow.

Despite the advantages listed here, we can highlight some precautions that must be taken. It is highly recommended to assess the anatomical plausibility of the marked point. The use of this technique should not be done without the detailed preoperative study of 2D multiplanar images. We must also emphasize some of its limitations. This method does not seem to be useful for infratentorial lesions. Peculiar anatomy of the brainstem and posterior fossa makes point localization on the surface less important. It must be considered that the need for many target points can make the technique too laborious and time cosuming. Besides, it does not provide intraoperative navigation; other resources must be associated for this purpose. The majority of cases were performed without neuronavigation systems. It would be recommended additional studies systematically measuring app error using neuronavigation as gold standard.

## Conclusions

Surgical planning with the aid of the NeuroKeypoint app is feasible and reliable. It has enabled neurological surgeries by craniotomy and trepanation in an accurate, precise, and less invasive manner.

## Data Availability

The datasets analyzed during this study are available on request. Code availability (software application or custom code): the code developed is available on request.
